# Prediction of clinical prognosis in cutaneous melanoma using an immune-related gene pair signature

**DOI:** 10.1080/21655979.2021.1924556

**Published:** 2021-05-28

**Authors:** Yu Yang, Xuan Long, Guiyun Li, Xiaohong Yu, Yu Liu, Kun Li, Xiaobin Tian

**Affiliations:** a Department of Emergency, The Second People’s Hospital of Guiyang, Guiyang, China; b Department of Obstetrics and Gynecology, The Affiliated Hospital of Guizhou Medical University, Guiyang, China; c Department of Orthopedics, Clinical Medical College, Guizhou Medical University, Guiyang, China

**Keywords:** Cutaneous melanoma, immune-related gene pairs, prognosis

## Abstract

Cutaneous melanoma (CM) is a malignant and aggressive skin cancer that is the leading cause of skin cancer-related deaths. Increasing evidence shows that immunity plays a vital role in the prognosis of CM. In this study, we developed an immune-related gene pair (IRGP) signature to predict the clinical prognosis of patients with CM. Immune-related genes from The Cancer Genome Atlas (TCGA) and the Gene Expression Omnibus (GEO) databases were selected to construct the IRGPs, and patients with CM in these two cohorts were assigned to low- and high-risk subgroups. Moreover, we investigated the IRGPs and their individualized prognostic signatures using Kaplan-Meier survival analysis, univariate and multivariate Cox analyses, and analysis of immune cell infiltration in CM. A 41-IRGP signature was constructed from 2498 immune genes that could significantly predict the overall survival of patients with CM in both the TCGA and GEO cohorts. Immune infiltration analysis indicated that several immune cells, especially M1 macrophages and activated CD4 T cells, were significantly associated with the prognostic effect of the IRGP signature in patients with CM. Overall, the IRGP signature constructed in this study was useful for determining the prognosis of patients with CM and for providing further understanding of CM immunotherapy.

## Graphical abstract

**Figure:**
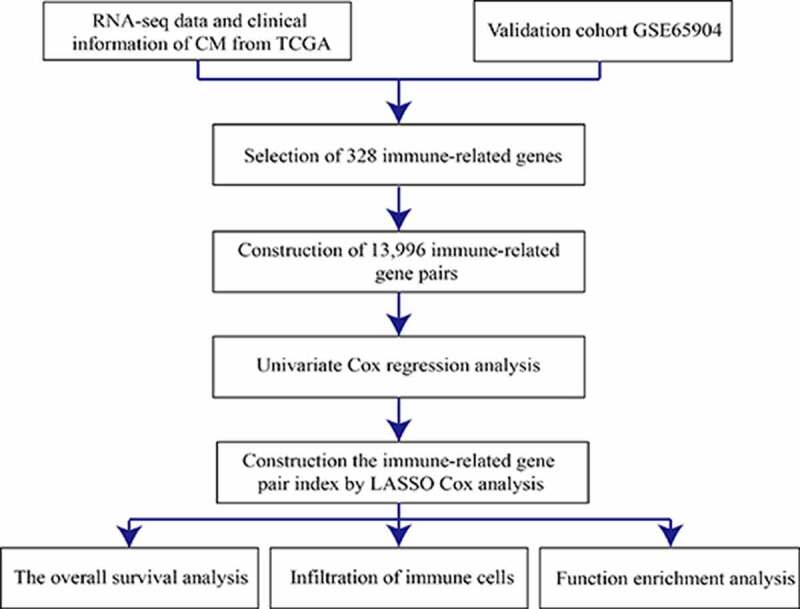

## 1. Introduction

Cutaneous melanoma (CM) arises from melanocytes and exhibits the most aggressive and complex characteristics of all skin cancers [1]. Its annual incidence continues to increase worldwide [2]. Owing to its strong tendency to metastasize, CM has been regarded as the primary cause of skin cancer-related deaths [3]. Current treatments for CM include surgical excision, immunotherapy, and therapy with kinase inhibitors [4,5]. Although CM at an early stage can be cured by surgery, the prognosis is still poor, with a 5-year survival rate of less than 10% once metastasis occurs [6]. The inherent complexity and heterogeneity of CM poses a challenge in predicting patient prognosis and response to therapy.

With the development of novel targeted immunotherapies, the discovery of biomarkers for prediction and prognosis has garnered considerable attention. In the past few years, the importance of the immune system in the development and progression of cancer has begun to be recognized [7,8]. Moreover, there is growing evidence that gene and immune regulation are involved both in the development of melanoma and in the improvement of prognosis [9]. Recently, several studies have constructed and validated prognostic signatures based on immune-related gene pairs (IRGPs) to evaluate the overall survival (OS) of various cancers, including osteosarcoma [10], pancreatic carcinoma [11], and ovarian carcinoma [12]. Components of the immune system play a crucial role in cancer progression. It has been reported that tumor-infiltrating immune cells are generally associated with tumor immune responses that can affect tumor growth, metastasis, and progression [13]. These findings raise questions regarding the analysis of progression in CM with IRGPs and the mechanisms underlying the complex interactions between CM and host immune response.

To develop an IRGP signature that is significantly related to the prognosis of patients with CM and to uncover the possible underlying mechanisms, we selected the immune-related genes (IRGs) from The Cancer Genome Atlas (TCGA) and the Gene Expression Omnibus (GEO) databases and constructed an IRGP signature using univariate Cox regression and LASSO analyses. Moreover, we investigated the IRGPs and their individualized prognostic signatures using Kaplan–Meier survival analysis, Cox analysis, and analysis of immune cell infiltration in CM. Furthermore, Gene Ontology (GO) and Kyoto Encyclopedia of Genes and Genomes (KEGG) pathway analyses were performed to determine the underlying mechanisms of immune-related signature genes in patients with CM. Gene set enrichment analysis (GSEA) was performed to identify the pathways that were most altered between the low- and high-risk subgroups. After a series analysis, we identified an IRGP signature with high sensitivity and specificity that could effectively predict the prognosis of patients with CM. Our study provides insight into the diagnostic and therapeutic targets for CM. Most importantly, our study provides a framework for the identification of a novel application of IRGs associated with specific tumors.

## 2. Materials and methods

### 2.1. Gene expression profiles and clinical data

The independent data included in this study were downloaded from a public database. The mRNA data of 471 CM samples and corresponding clinical information were obtained from the TCGA database (https://cancergenome.nih.gov/). All samples obtained from the TCGA platform were used as the training dataset. The RNA-seq expression dataset GSE65904, which includes a large number of CM patients (214 samples), was downloaded from the GEO database (https://www.ncbi.nlm.nih.gov/geo/) as the matched testing dataset that has been widely applied in previous studies [14,15]. All data were collected on 27 March 2020.

### 2.2. Gene information processing

For all collected datasets, the expression profile was converted from the probe level to the corresponding gene symbol based on the strength of each set of annotation files. The average gene expression level of the target gene, which was matched by multiple probes, was further explored to represent the expression value of a single gene. Only datasets with complete OS information were selected for the subsequent studies.

### 2.3. Construction of a prognostic IRGP signature

Based on previous studies on IRGPs [16], to investigate the prognostic immune characteristics of CM, 2498 IRGs were downloaded from the ImmPort database (https://www.immport.org) [17], which contains a series of IRGs such as macrophages and genes relating to the T-cell receptor signaling pathway, B-cell antigen receptor signaling pathway, and natural killer cell cytotoxicity. The expression levels of the IRGs in all cohorts were analyzed using the ‘Limma’ package of the Bioconductor in R statistical software. IRGs that were expressed jointly in two cohorts and with a relatively high variation (median absolute deviation > 0.5) were subjected to subsequent pairwise comparisons [18], from which the scores for each IRGP were generated. The scores were calculated as follows: if the expression level of IRG1 was larger than that of IRG2, the score was 1; otherwise, the score was 0. Next, IRGPs with constant values (0 or 1) were removed from all individual datasets included in the meta-dataset, and the remaining IRGPs were considered candidate IRGPs.

The candidate IRGPs selected for the prognostic model were filtered using a Cox proportional hazards regression model combined with the log-rank test to evaluate the relationship between candidate IRGPs and sample OS in the training cohort (p < 0.001). To build the IRGP index (IRGPI), we used the LASSO Cox proportional hazards (‘glmnet’ R package) [19] with 10-fold cross validation to minimize the risk of overfitting. Forty-one gene pairs were finally selected to construct the prognostic model. The IRGPI was further used to calculate the risk score of each sample, which was then used to divide patients with CM into low- or high-risk subgroups. The optimal cutoff was determined using a time-dependent receiver operating characteristic (ROC) curve (‘survivalROC’ R package) [20] of the training set at 5 years.

### 2.4. Validation of the prognostic model

Univariate and multivariate Cox proportional hazards regression analyses were performed based on the OS of the patients and the IRPGI risk score for each cohort. A Kaplan–Meier plot was constructed to assess the difference in OS between the low- and high-risk subgroups.

### 2.5. Infiltration of immune cells in the tumor

CIBERSORT (http://cibersort.stan ford.edu/) [21] was used to predict the infiltration levels of immune cells in the low- and high-risk groups. We calculated the expression matrix of 22 immune cells in each tumor sample using CIBERSORT, and the *p*-value was calculated to determine the accuracy of the prediction. Wilcoxon test was used to determine the correlation between immune cell infiltration and the risk scores of the samples. Correlations were visualized using boxplots and radar plots.

### 2.6. Function enrichment analysis

GSEA, a powerful analytical method for interpreting gene expression data [22], was used to analyze the significant functional pathways of our prognostic immune signature in the TCGA dataset. The log2 fold change in gene expression was calculated between the high- and low-risk subgroups, with the pathways involved in this analysis being acquired from the Molecular Signature Database (version 7.2; C2 databases, http://www.gsea-msigdb.org/gsea/downloads.jsp). GSEA was performed using the ‘fgsea’ R package and permutated 10,000 times. An FDR-adjusted *p*-value of <0.002 was considered statistically significant.

To further understand the underlying mechanisms of our identified immune-related signature genes in CM patients, GO and KEGG pathway analyses were performed using the ‘ClusterProfiler’ package version 3.6.3 in R software as previously described [23]. Three terms, namely, biological process (BP), cellular component (CC), and molecular function (MF), were used in the GO analyses. *p* < 0.05 and FDR < 0.05 were considered statistically significant.

## 3. Results

To identify a novel application of IRGs in CM, an IRGP signature with high sensitivity and specificity that could effectively predict the prognosis of patients with CM was constructed in this study. The correlation between immune cell infiltration and IRGPI was also examined.

### 3.1. Construction of a prognostic IRGP signature

**Figure 1.**
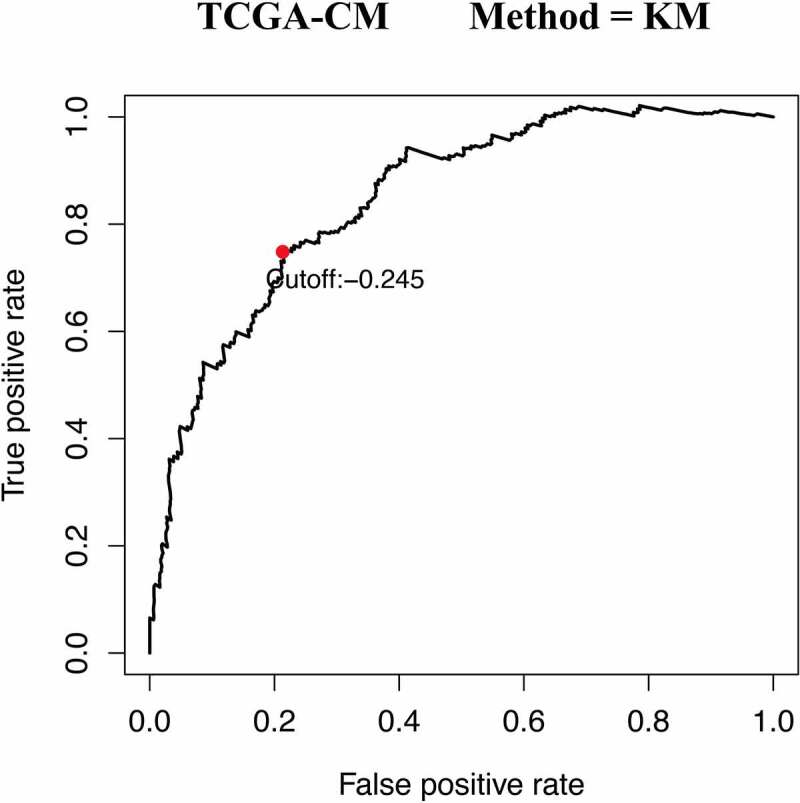
Time-dependent ROC curve for IRGPI at 5 years in the TCGA-CM cohort

A total of 471CM samples were collected from the TCGA database, of which 214 tumor samples from the GSE65904 cohort were included in this study. Of the 2,498 IRGs downloaded from the ImmPort database, 328 IRGs were measured in both mate-training and mate-testing sets. Using these 328 selected IRGs, 13,996 IRGPs were constructed. We then performed Cox proportional hazards regression analyses for the OS of the meta-training cohort, thereby obtaining 2,215 prognostic IRGP candidates. Next, LASSO Cox proportional regression analysis was used to randomly remove gene pairs with high similarity. After a series of screenings, 41 IRGPs were selected to construct the final prognostic signature (Table 1). The IRGPI was then constructed to calculate the risk score. Based on the time-dependent ROC curve analysis, the optimal cutoff value of the IRGPs was determined to be −0.245 (Figure 1).

**Table 1.** Prognostic signature information about 41 constructed IRGPs

IRG 1	IRG 2	Coefficient	IRG 1	IRG 2	Coefficient
CD8A	CETP	−0.07	IDO1	TGFB3	−0.03
FCER1G	PTGDS	−0.01	IRF1	SEMA6A	−0.21
HLA-DQB1	S100A8	−0.16	IRF1	MET	−0.11
HLA-DQB1	CDH1	−0.02	JUN	SEMA6A	−0.06
HSPA2	CX3CL1	0.15	GNLY	SEMA4A	−0.03
HSPA2	FGFRL1	0.05	BPHL	CMTM8	0.01
CXCL14	RSAD2	0.02	MARCO	PLAUR	−0.02
CXCL1	ITK	0.02	CCL28	TRIM22	0.03
CCL8	APLNR	−0.17	IRF7	PIK3CD	−0.06
CCL8	TUBB3	−0.04	PDGFRB	TNFSF13B	0.02
S100A9	STAT1	0.04	GBP2	LTBR	−0.13
S100A9	RARRES3	0.01	CCR1	RAC3	−0.01
S100A8	IFIH1	0.10	CD79B	LTB	0.02
SLC22A17	CRABP2	−0.11	RAC3	SEMA3B	0.04
APOBEC3G	ANGPTL2	−0.01	RAC3	FGFRL1	0.04
PLAU	RARRES3	0.01	CD72	EDNRA	−0.06
NOX4	EDNRA	−0.12	IFITM1	GPI	−0.001
CRABP2	CYBB	0.09	SEMA3C	SEMA6A	−0.05
CYBB	CD79B	−0.01	TNC	CLEC11A	−0.07
IFIH1	BMPR2	−0.01	IL17D	SCG2	0.10
IDO1	EDNRA	−0.03

### 3.2. Validation of the prognostic model

**Figure 2.**
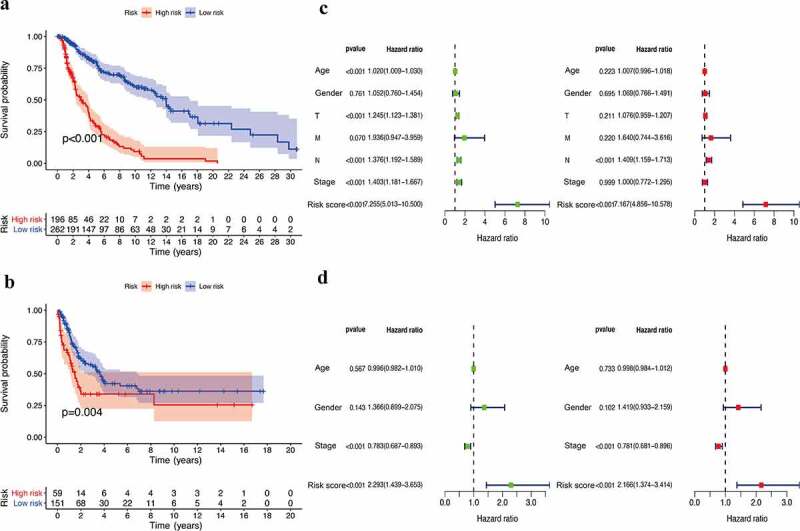
Efficacy evaluation of constructed IRGPI risk model. (a) Kaplan-Meier survival curve of TCGA cohort. (b) Kaplan-Meier survival curve of GSE65904 cohort. Univariate and multivariate Cox regression analysis of the clinicopathological features in TCGA (c) and GSE65904 (d) cohorts

Based on the optimal cutoff of the risk scores, the training set was divided into low- (n = 262) and high-risk (n = 196) groups. The survival analysis data showed that the IRGPI had a good prognosis for the training dataset and that the patients in the high-risk group had a significantly lower survival probability than those in the low-risk group (p < 0.001) (Figure 2(a)). Similar to that observed in the training group, a higher IRGPI was associated with a lower OS in the GSE65904 cohort (p = 0.004) (Figure 2(b)). Furthermore, univariate Cox analysis showed that age, tumor stage, and risk score had prognostic effects in the training database. However, only the IRGPI could be regarded as an independent prognostic parameter in patients with CM, as determined using multivariate Cox analysis (p < 0.001) (Figure 2(c)). To verify the prognostic signature in the test dataset, univariate and multivariate Cox analyses were also performed in the GSE65904 cohort, and the results suggested that the IRGPs showed high connectivity with regard to predicting the OS of patients with CM (Figure 2(d)).

### 3.3. Infiltration of immune cells in CM

**Figure 3.**
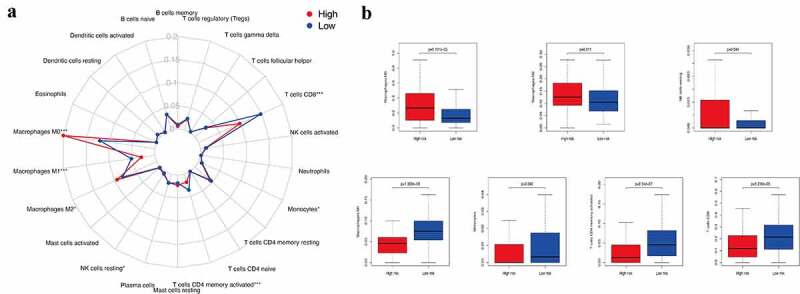
The infiltration level of immune cells in the constructed IRGPI risk model. (a) Summary of the 22 tumor-infiltrating immune cells’ abundance estimated by CIBERSORT analysis. *p*-values are based on t-test (*p< 0.05, ***p< 0.001). (b) The boxplot shows the distribution of specific immune cells in two risk subgroups from the TCGA cohort

Tumor infiltration by immune cells is associated with prognosis [24]. In this study, we used CIBERSORT to analyze the expression of 22 tumor-infiltrating immune cells in the TCGA cohort across the low- and high-risk subgroups (Figure 3(a)). Our results showed that M0 macrophages (p = 9.101e−05), M2 macrophages (p = 0.011), and resting NK cells (p = 0.044) were significantly enriched in the high-risk subgroup. M1 macrophages (p = 1.069e−09), monocytes (p = 0.040), memory-activated CD4 T cells (p = 8.54e−07), and CD8 T cells (p = 5.296e−05) were clustered in the low-risk subgroup (Figure 3(b)). Thus, both the tumors lacking memory CD4 T cells, M1 macrophages, monocytes, and CD8 T cells and those with an increased number of M0 and M2 macrophages and NK cells were associated with poor prognosis of CM in clinical practice.

### 3.4. Functional enrichment analysis

**Figure 4.**
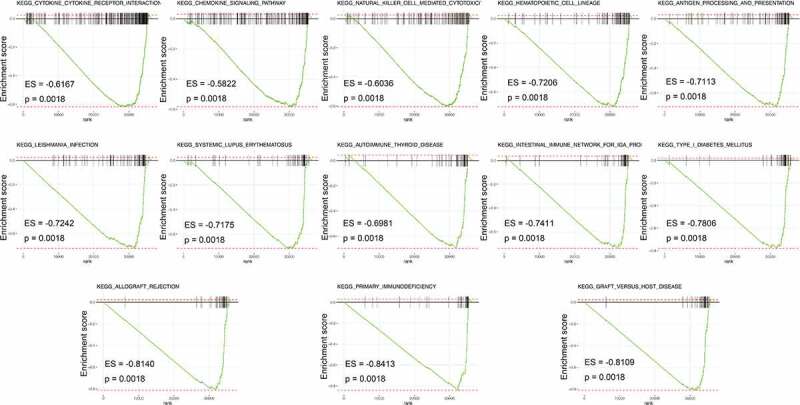
Gene set enrichment analysis (GSEA) between high- and low- IRGPI risk subgroups. The results show that 13 cancer hallmark gene sets are enriched in the low IRGPI risk subgroup (FDR < 0.002)

**Figure 5.**
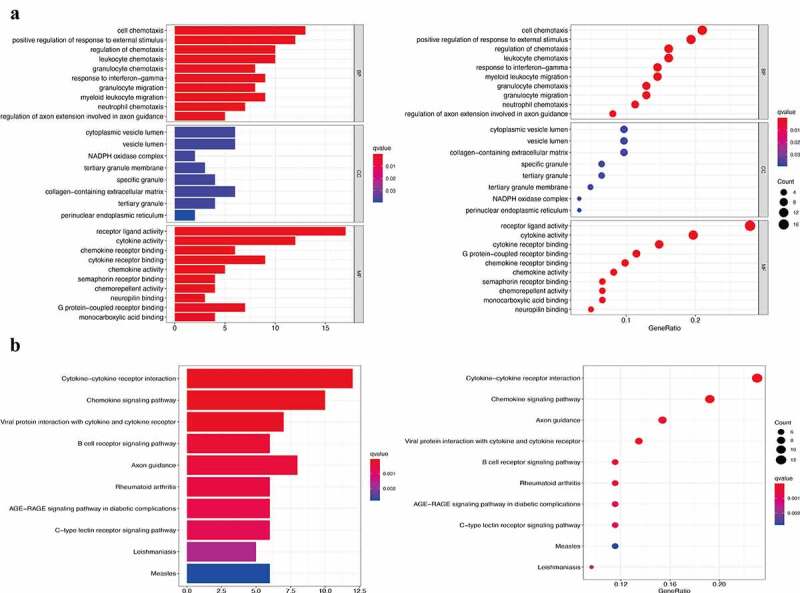
Functional enrichment analysis of 82 immune signature genes. (a) Top 10 classes of GO enrichment terms in biological process (BP), cellular component (CC), and molecular function (MF). (b) Top 10 classes of KEGG enrichment terms. In each bubble plot, the size of the dot represents the number of enriched genes

Using GSEA, all expressed genes were incorporated to investigate the enrichment of the functional pathways between the low- and high-risk subgroups. In total, 13 hallmark gene sets were associated with the IRGPI signature (FDR < 0.002), including those responsible for ‘cytokine-cytokine receptor interaction,’ ‘chemokine signaling pathway,’ and ‘natural killer cell mediated cytotoxicity’ (Figure 4). This, in turn, revealed the hallmark gene sets that were significantly associated with the IRGPI signature in CM progression, thereby predicting the OS of patients with CM. The GO analysis results (Figure 5(a)) showed that 82 IRGs were primarily involved in critical BPs, such as cell chemotaxis, positive regulation of responses to external stimuli, and regulation of chemotaxis. These IRGs were also found to be enriched in response to the vesicle lumen, NADPH oxidase complex, and tertiary granule membrane in the CC category and in receptor-ligand activity, cytokine activity, and chemokine receptor binding in the MF category. Moreover, KEGG enrichment analysis indicated that the selected IRG was significantly associated with the pathways of cytokine-cytokine receptor interaction, chemokine signaling pathway, and viral protein interaction with cytokines and cytokine receptors (Figure 5(b)).

## 4. Discussion

The prognosis of patients with CM, like in many types of cancer, relies on early detection, diagnosis, and treatment. Recently, various novel technologies have been applied in tumor analysis, such as fungal-derived materials and smartphone-based portable electrochemical biosensing systems. Fungal cultures, such as statins and penicillin, are widely used in healthcare. In recent decades, the bioactive compounds of these cultures have also been shown to have anticancer potential and drug delivery function [25]. The smartphone-based biosensing system is another new method that can realize efficient, low-cost, and portable on-site diagnosis by detecting cancer biomarkers, such as circulating microRNAs, in body fluids [26]. Furthermore, to augment the continuous emergence of new therapeutic targets, current research is focused on the discovery of prognostic biomarkers related to the measurable diagnostic indicators currently used to assess disease risk [27,28]. With the development of novel targeted immunotherapies, the discovery of biomarkers for prediction and prognosis has attracted much attention. In the last few years, the importance and participation of the immune system in the development and progression of cancer have begun to be recognized [7,8]. Components of the immune system play a crucial role in cancer progression. Studies have found that tumor-infiltrating immune cells are generally associated with tumor immune responses that can affect tumor growth, metastasis, and progression [13]. These findings facilitate discussions on using IRGPs to analyze tumor progression in CM, as well as on the determination of the mechanisms of complex interactions between tumors and the host immune response.

In the present study, we developed and validated a 41-IRGP signature to predict the OS of patients with CM. Patients were stratified into low- and high-risk subgroups based on the IRGP signature. Results of the survival analysis indicated that the IRGPI had a good prognosis for the training dataset and that the patients in the high immune risk group had a smaller survival probability than those in the low-risk group. Similar to that in the training group, a higher IRGPI was associated with a lower OS in the GSE65904 cohort. Results of the Cox proportional hazard regression analyses suggest that the IRGPI was an independent prognostic factor in patients with CM.

Various studies have confirmed that the immune response is significantly associated with tumor initiation and progression and that it plays a critical role in malignant cancer occurrence [29,30]. In this study, several functional pathways involved in the immune response were associated with the IRGPI signature, which includes natural killer cell-mediated cytotoxicity, antigen processing and presentation, immune network for IgA production, and primary immunodeficiency. Thus, to further explore the relationship between immune cell expression and IRGPs, CIBERSORT analysis was performed in this study. The results demonstrated that both a lack of memory CD4 T cells, M1 macrophages, monocytes, CD8 T cell infiltration and an increase in the numbers of M0 and M2 macrophages and NK cells were associated with poor prognosis in patients with CM. These results are largely consistent with those of previous studies on immune cells, such as M2 macrophages, which have been confirmed to be associated with poor prognosis in several cancers [31]. To a certain extent, these results explain how the IRGP risk model can accurately predict the prognosis of patients with CM. We also observed that some pathways, such as the cytokine-cytokine receptor interaction pathway, chemokine signaling pathway, and B cell receptor signaling pathway, were significantly related to our identified IRGs. Of these, the cytokine-cytokine receptor interaction pathway was the most significantly enriched, which may influence the prognosis value of IRGs in patients with CM. Previous studies have shown that cytokine-cytokine receptor interaction is significantly associated with various CM processes, such as exercise therapy and CM metastasis-related microRNA and mRNA expression. This interaction may play a fundamental role in modulating tumor metastasis, which is a critical factor affecting the OS of patients with CM [32–34]. Further, the chemokine [35] and B cell receptor signaling pathways [36,37] are also important factors that affect the prognosis of patients with CM. These results may provide novel research ideas and a basis for future studies on the role of IRGs in patients with CM.

However, our study has some limitations. First, owing to high cost and demand, the prognostic signatures based on gene expression proﬁles generated by RNA-seq or microarray platforms are difﬁcult to popularize for use in clinical settings. Thus, more conveniently available and inexpensive methods of detecting the expression of IRGs are urgently needed. Second, our immune prognostic signature was developed using retrospective studies. Thus, prospective cohort studies are required to further validate our results. Finally, the signature was constructed using RNA-seq and microarray expression data. It should be further evaluated using RT-PCR and IHC prior to use in a clinical setting.

## 5. Conclusion

In summary, we systematically investigated the prognostic value of immune-related gene pair signatures. The robust IRGP signature can effectively predict the clinical prognosis of patients with CM, and our study provides further understanding of the role of our prognostic signature in the development of CM. Most importantly, our study provides a framework for identifying a novel application of IRGs associated with specific tumors.

## Supplementary Material

Supplemental Material

## Highlights

1. IRGPI is associated with overall survival of cutaneous melanoma.
2. IRGPI can be regarded as an independent prognostic parameter of cutaneous melanoma.
3. IRGPI in predicting cutaneous melanoma prognosis is significantly associated with multiple immune-cell infiltration.
