# Supplementary figures and images for: Prediction of clinical prognosis in cutaneous melanoma using an immune-related gene pair signature
Source: Bioengineered. 2021 May 28;12(1):1803–12. doi: 10.1080/21655979.2021.1924556 (PMC8806557; doi:10.1080/21655979.2021.1924556)

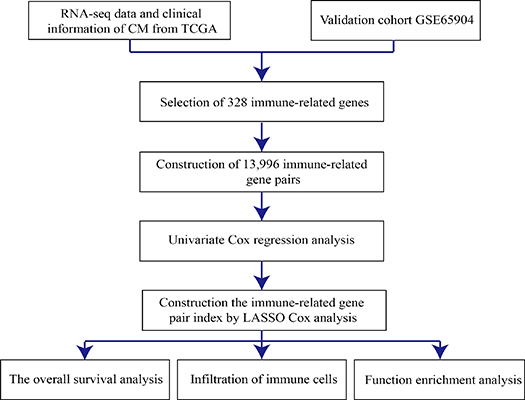

Supplement: Supplemental Material [file KBIE_A_1924556_SM5320.jpg]
